# Decellularized Nerve Scaffold in a Rat Model of Extensive Peripheral Nerve Damage

**DOI:** 10.17691/stm2025.17.6.02

**Published:** 2025-12-29

**Authors:** T.V. Rusinova, R.A. Vinogradov, A.S. Asyakina, G.P. Chuprynin, A.A. Fomenko, E.A. Solop, K.I. Melkonyan

**Affiliations:** PhD, Researcher, Central Research Laboratory; Kuban State Medical University, 4 Mitrofana Sedina St., Krasnodar, 350063, Russia; MD, DSc, Associate Professor, Professor, Department of Surgery No.1; Kuban State Medical University, 4 Mitrofana Sedina St., Krasnodar, 350063, Russia; Junior Researcher, Central Research Laboratory; Kuban State Medical University, 4 Mitrofana Sedina St., Krasnodar, 350063, Russia; Research Laboratory Assistant, Central Research Laboratory; Kuban State Medical University, 4 Mitrofana Sedina St., Krasnodar, 350063, Russia; Research Laboratory Assistant, Central Research Laboratory; Kuban State Medical University, 4 Mitrofana Sedina St., Krasnodar, 350063, Russia; Research Laboratory Assistant, Central Research Laboratory; Kuban State Medical University, 4 Mitrofana Sedina St., Krasnodar, 350063, Russia; MD, PhD, Associate Professor, Head of Central Research Laboratory; Kuban State Medical University, 4 Mitrofana Sedina St., Krasnodar, 350063, Russia

**Keywords:** peripheral nerve regeneration, decellularization, autograft, nerve scaffold, decellularized matrix

## Abstract

**The aim of the study** was to develop a modified protocol for rat sciatic nerve decellularization and evaluate its efficacy in a rat model of an extensive peripheral nerve defect.

**Materials and Methods:**

The study described a modified detergent-enzymatic decellularization protocol to create a decellularized nerve scaffold. A decellularization process (24 h) included the sequential treatment of a rat sciatic nerve with the solutions of trypsin–versene, 1% Triton X-100, 4% sodium deoxycholate, phosphate-buffered saline, and pancreatic DNase I. We modelled an extended defect of the sciatic nerve (15±2 mm), and Wistar rats were implanted with autografts or decellularized nerve scaffolds. The nerve recovery was assessed on day 90 after implantation using an immunohistochemistry analysis of the total count of nerve fibers, intact motor fibers, and myelinated fibers.

**Results:**

The results of the histological examination and DAPI staining showed the complete destruction and washing of nuclear material to preserve the nerve structure after decellularization. The DNA content in the decellularized scaffolds was 48.17±4.25 ng/mg of tissue, while in the native nerve it was 221.51±1.36 ng/mg. The analysis of the tissue response to the decellularized scaffold subcutaneously implanted demonstrated the absence of macrophages. Histology revealed a moderate number of intact nerve fibers in the orthotopically implanted decellularized scaffold (835.6 [804.2; 866.0] per 1 mm^2^ of tissue) compared to the autograft (1284.1 [1190.5; 1316.0] per 1 mm^2^ of tissue). The decellularized scaffold implantation resulted in an increase in the number of small myelinated fibers and the restoration of motor and sensory nerve fibers.

**Conclusion:**

The use of detergent-enzymatic decellularization of the rat sciatic nerve demonstrated high efficacy, which was confirmed by the absence of nuclear material with the nerve histological structure preserved. The presence of the sufficient number of Schwann cells 3 months after implantation and the perineurium formation are the positive characteristics when assessing the nerve decellularization protocol efficacy. Thus, the decellularized nerve scaffold is a promising replacement of autografts to treat extensive defects of peripheral nerves.

## Introduction

The regeneration of peripheral nerve defects is still a multifaceted issue, despite the development of novel biomaterials and treatment techniques [1]. Nerve grafts are used to restore nerves in case a defect is over 5 mm, and it is impossible to suture directly without tension. Nerve autografting remains the basic surgical technique to treat the extensive damage of peripheral nerves in clinical practice aimed at restoring their functionality. However, the method has its disadvantages, including the shortage of donor materials, the difficulty in obtaining donor materials, and a long-term period of postoperative restoration [2]. Decellularized nerve scaffolds appear to be an effective approach to treat damaged peripheral nerves; it enable to prevent autografting shortcomings [3]. Such scaffolds perform a supporting function and stimulate the targeted regeneration of a nerve [4]. The findings of the recent studies carried out both *in vitro* and *in vivo* have emphasized a stimulating effect of the decellularized extracellular matrix (ECM) on restorative processes. ECM components have a reparative potential and appear to have a positive effect on axonal recovery rate [5–7]. The process of obtaining allogenic or xenogenic tissue scaffolds based on ECM includes different physical, chemical, and enzymatic processing techniques, however, the lack of standardized protocols for producing decellularized tissues for regenerative medicine is still challenging [8, 9]. Optimization of the protocol for decellularized nerves will provide obtaining the most appropriate material for implantation and stimulation of regenerative processes when treating the peripheral nerve damage.

**The aim of the study** was to develop a modified protocol for rat sciatic nerve decellularization and evaluate its efficacy in a rat model of an extensive peripheral nerve defect.

## Materials and Methods

### Material preparation

The experiments were carried out on mature male Wistar rats weighing 190±10 g based at Kuban State Medical University (Russia). Management, care, marking, and other procedures of the laboratory animals were performed according to the European Convention for the Protection of Vertebrate Animals used for Experimental and other Scientific Purposes (Strasburg, 1986) and were approved by the local ethics committee of Kuban State Medical University (protocol No.118 dated March 3, 2023). In the course of experiments, a rat sciatic nerve was isolated (the size of the samples was 15±2 mm) and placed into an Eppendorf tube with phosphate-buffered saline and antibiotic (1% penicillin–streptomycin solution) for further freezing at –80°С. After that, the sciatic nerve samples were subjected to cyclic detergent-enzymatic decellularization.

### Decellularization techniques

The samples underwent the solution treatment in the ratio of the sample weight (g) to the solution volume (ml) — 1:10. The unfrozen nerves were treated with trypsin–versene solution (BioloT, Russia) at 37°С in a shaker incubator at 100–150 rpm for 6 h, with the solutions being changed every 2 h. Then the samples had two cycles of successive exposure by 1% Triton X-100 solution (Scharlab, Spain) for 3 h in a shaker at 100–150 rpm and 4% sodium deoxycholate solution (Sigma-Aldrich, USA) for 3 h at room temperature. The samples were washed with distilled water after each solution for 5–20 min followed by treating with swine pancreatic DNase I (2000 IU/200 ml of calcium/magnesium phosphate buffer; Sigma-Aldrich, USA) for 4 h at 37°С in a shaker incubator at 100–150 rpm. The obtained samples were checked for decellularization quality using hematoxylin and DAPI staining and by counting the DNA amount compared with the native nerve.

### Quantitative DNA test

DNA quantification was performed on a spectrophotometer NanoDrop ND-1000 (Thermo Fisher Scientific, USA) using a reagent kit (ExtractDNA Blood & Cells; Eurogen, Russia) in accordance with the manufacturer’s protocol.

### Histological analysis

All samples were kept in 10% neutral buffered formalin solution for 24 h. Then the samples were embedded in paraffin according to a standard technique using a histoprocessor TP1020-1 (Leica, Germany). Paraffin blocks were made using a package unit EG1150H (Leica, Germany), after that, the slides were prepared on a rotor microtome RM2235 (Leica, Germany). The preparations, 5 μm thick, were placed on microscopic slides, deparaffinized in xylol and in a calibrated series of ethyl alcohols. The next stage included the samples being hematoxylin and eosin stained, washed in flowing water, fixed under cover glasses using the synthetic embedding medium (Diapath, Italy), and photographed in three fields of vision using Olympus CX 41 microscope (Olympus, Japan). The images and data were analyzed using the software CellSens Entry (Olympus, Japan).

### DAPI staining

The histological sections (4–5 μm) of the native nerve and the decellularized scaffold of the peripheral nerve were fixed in 4% formaldehyde for 10 min. After that the sections were applied with DAPI (dilution 1:1000; Sigma-Aldrich, USA), covered by a cover glass, and demonstrated the characteristic staining under a fluorescent microscope (blue filter) Olympus IX 51 (Olympus, Japan). The cell nuclei fluoresced bright blue.

### Material cytotoxicity assessment

To determine the cytotoxicity of the decellularized nerve there was analyzed the viability of the cells in the presence of the obtained material using LIVE/DEAD test (LIVE/DEAD Cell Imaging Kit; Thermo Fisher Scientific, USA). We used the line of human dermal fibroblasts DF-1 received from Russian Collection of Vertebrate Cell Cultures in Institute of Cytology, Russian Academy of Science. The complete medium with no decellularized nerve added was used as a control. Relative fluorescence was rated based on three images randomly taken at 40-fold magnification. Fluorescent light was imaged using the software Olympus cellSens Entry (Olympus, Japan).

### Subcutaneous implantation

Subcutaneous implantation was performed at the animal withers along the spine midline under isoflurane anesthesia (induction 2–5%, flow 0.25–4.0%; MIRALEK, China). The experimental animals (n=8) were randomly divided into 2 groups. Group A animals were implanted with a decellularized scaffold of the peripheral nerve (15±2 mm), and group B animals — a native sciatic nerve (13±2 mm). The decellularized scaffold of the peripheral nerve was fixed in the muscular-fascial bed, the skin being sutured using Ethilon 3-0 (Ethicon, USA). After surgery each rat was injected with cefovecin at a dose of 6.4 mg/kg and ketoprofen — 12.8 mg/kg. Two weeks after implantation the animals were sacrificed being intramuscularly injected with zolazepam (15 mg/kg) and tiletamine (15 mg/kg). The samples of decellularized and native nerves with surrounding tissues were explanted; the sections were prepared for a histological analysis.

### Implantation

Experimentally, the peripheral nerve defect in Wistar rats was treated under anesthesia with isoflurane. The preoperative preparation involved depilation and the rat thigh skin treatment with 70% ethanol to prevent the wound inflammation and infection. The rats (n=12) were randomly divided into 2 groups. The peripheral nerve injury was modeled by dissecting the sciatic nerve, 15±2 mm long, above the bifurcation. Group 1 consisted of the controls (n=6), the nerve was rotated through 180° around the longitudinal axis and implanted using epineural sutures; group 2 was experimental (n=6), the decellularized scaffold of the peripheral nerve was implanted orthotopically. The defect was sutured using 5–6 epineural stitches with Promilen 8-0 (Ethicon, USA), the skin — 5 simple interrupted stitches using Ethilon 3-0 (Ethicon, USA). Postoperatively, each rat was injected with cefovecin at a dose of 6.4 mg/kg and ketoprofen — 12.8 mg/kg. The postoperative suture was treated with aerosol Terramycin (Zoetis, USA). On day 90 the rats were sacrificed being intramuscularly injected with zolazepam (15 mg/kg) and tiletamine (15 mg/kg). The decellularized scaffolds of the peripheral nerves and autografts were isolated and divided into proximal, medial, and distal segments relative to the implantation site, followed by their being subjected to histological and immunohistochemical analyses.

### Immunohistochemical analysis

The study involved the following antibodies: goat antibodies to choline acetyltransferase (ChAT, 1:100; Millipore, USA); rabbit antibodies to neurofilaments (NF, 1:200; Novus, USA), rat antibodies to myelin basic protein (MBP, 1:50; Abcam, Great Britain). We assessed the number of undamaged motor fibers by choline acetyltransferase stained (ChAT); the number of nerve and myelinated fibers by the staining of neurofilament (NF) and myelin basic protein (MBP), respectively. After that we applied videospecific conjugated antibodies with high Cy2 or Cy3 content (Jackson ImmunoResearch, USA). The change degree of the nerves was estimated by a semiquantitative method using the relative area of myelinated fibers calculated using a digital morphometric technique on samples. For staining ChAT we used biotinylated secondary antibodies and Cy2-conjugated streptavidin (Jackson ImmunoResearch, USA). Digital images were taken using Adobe Photoshop (Adobe Systems, USA). The number of ChAT or NF-positive neurons, as well as the diameters of MBP-positive fibers in three sections from each animal, were studied using the software Image-Pro Plus (Media Cybernetics, USA). Fixed sections of 200×200 μm were analyzed, there being taken 3–4 measurements from each section. Immunohistochemical staining was carried out in the laboratory Patho Logica (Israel).

### Statistical analysis

The findings were processed using MS Excel (version 6.0), GraphPad Prism for Windows (version 6.04; GraphPad Software, USA). The normality of data distribution was determined using Shapiro–Wilk test. Immunohistochemistry results and determining the number of DNA were analyzed using Student t-test and represented in the form М±SD, where М — arithmetic mean, SD — standard deviation. The histological analysis findings were studied using Mann–Whitney rank test, and represented as a median with the first and third quartiles (Me [Q1; Q3]). The differences were considered significant if p<0.05.

## Results

**Figure 1.**
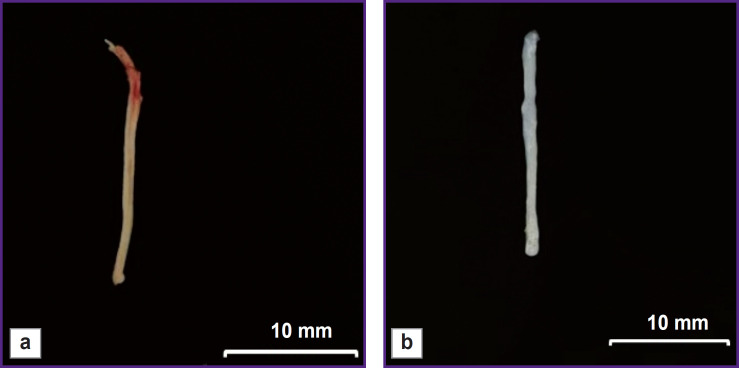
Appearance: (a) native nerve; (b) decellularized scaffold of the peripheral nerve

The main advantage of the peripheral nerve decellularization is the possibility to obtain non-immunogenic biological material, which preserves the tissue functional structure, although it has no cellular components causing an immune response in implantation. A decellularized scaffold was a white scaffold with frayed edges, had the smooth surface and looser structure compared to the native nerve samples (Figure 1).

**Figure 2.**
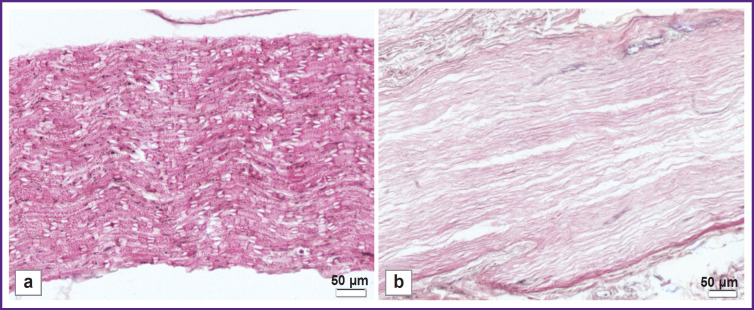
Histological staining of the sample structure: (a) native nerve; (b) decellularized scaffold of the peripheral nerve. Hematoxylin and eosin staining; 200×

The changes in the decellularized scaffold structure were assessed using histochemical staining compared to the native nerve. Hematoxylin and eosin staining demonstrated the preservation of the general histological nerve architecture after the cell components being removed (Figure 2).

**Figure 3.**
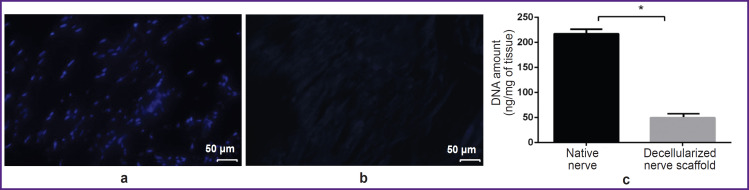
Evaluation of the obtained decellularized nerve: (a) cytotoxicity assessment of the native nerve; (b) cytotoxicity assessment of the decellularized scaffold of the peripheral nerve; DAPI staining; 200×; (c) quantitative analysis assessment of the decellularized scaffold DNA; * the differences of the values are significant in relation to the native nerve, p<0.05

The decellularized nerve scaffold samples had no cellular nuclei that confirmed the decellularization efficiency. DNA content in the native samples was 221.51±1.36 ng/mg of tissue, in the decellularized nerve scaffold — 48.17±4.25 ng/mg of tissue that was 4.59 times lower than the native nerve characteristics (p<0.05) (Figure 3).

As follows from LIVE/DEAD test, in the control well the viability of DF-1 line cells was 87.60±5.41%, and in the presence of the material under study — 83.2±4.6%. It indicated the absence of toxic effects of the decellularized nerve on the cultured cells. Materials are considered biocompatible if the viability percentage is over 70% in accordance with GOST ISO 10993-5—2011 criteria [10].

**Figure 4.**
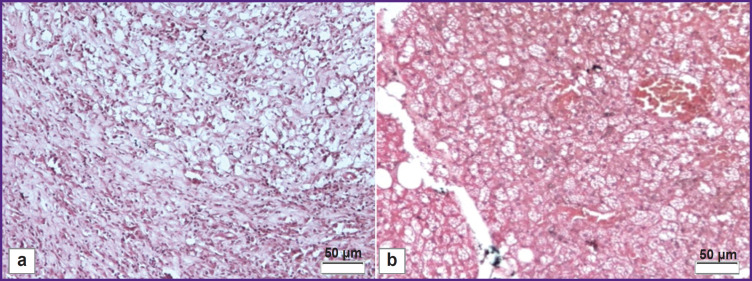
Histological staining of the subcutaneous implantation on day 14: (a) native nerve; (b) decellularized scaffold of the peripheral nerve. Hematoxylin and eosin staining; 200×

Subcutaneous implantation demonstrated no inflammatory response to the decellularized scaffold. The histological assessment showed no macrophages and neutrophils in the area between the decellularized scaffold and the adjoining tissue (Figure 4 (b)). Meanwhile, the native nerve implantation demonstrated significant lympho-macrophagic infiltration (Figure 4 (a)).

**Figure 5.**
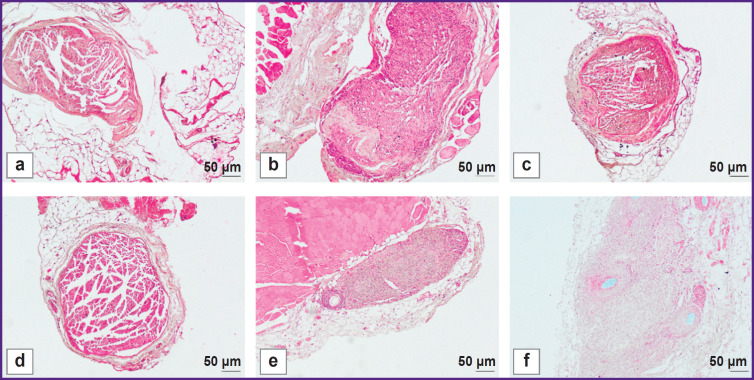
Histological evaluation of the peripheral nerve regeneration efficiency on day 90 after implantation: (a)–(c) group 1 (autograft); (d)–(f) group 2 (decellularized scaffold of the peripheral nerve); (a), (d) proximal nerve segment; (b), (e) medial nerve segment; (c), (f) distal nerve segment. Hematoxylin and eosin staining; 200×

The visual examination of the animals in all experimental groups revealed the decreased volume of the posterior limbs and the gait impairment related to the limb deformity and phalange contracture. 90 days after implantation, the experimental group animals were found to have the nerve fibers prevailing in the medial nerve segment according to a histological analysis (Figure 5). The assessment of the decellularized nerve scaffold samples in these segments showed the moderate number of intact nerve fibers, which was 835.6 [804.2; 866.0] per 1 mm^2^ of tissue, the fiber diameter — 10.1 [8.2; 12.4] μm. There was observed the formation of the marked perineurium and the decreased amount of loose connective tissue between the tissue fibers. The histological evaluation results of nerve fiber restoration in autograft implantation were the best among all experimental groups. In autografting in medial segments of the samples, there were recorded 1284.1 [1190.5; 1316.0] intact fibers per 1 mm^2^ of a transverse section, their diameter was 10.2 [9.7; 11.0] μm. In the area between the implanted materials, there was found the insignificant amount of loose endoneurium; the perineurium was a dense connective tissue layer.

An immunohistochemical analysis showed the significant restoration of motor nerve fibers in the medial graft segment in the experimental group compared to the control one (see the Table).

**Table:** Immunohistochemical assessment of the rat sciatic nerve repair in experimental groups

Group	Segment	NF	ChAT	ChAT/NF·100%	MBP, 1–6 μm in diameter	MBP, 7–20 μm in diameter
Autograft	Proximal	410.5±5.5	82.3±2.6	20.0±0.9	72.8±2.9	124.8±6.9
	Medial	315.3±20.2	53.8±2.4	17.1±0.8	102.8±4.4	48.5±2.7
	Distal	165.7±6.2	33.0±2.0	19.9±0.9	102.0±2.6	5.5±0.2
Decellularized nerve	Proximal	409.0±31.2	84.7±2.1	20.7±1.6	58.0±3.6	93.7±2.6
	Medial	276.7±19.6	113.0±7.0*	40.8±0.4*	158.3±4.7	26.0±1.5*
	Distal	146.0±4.7	19.3±2.4*	13.2±1.5	76.3±2.1*	21.3±1.8*

N o t e: there is indicated the average number of intact motor fibers (ChAT-positive fibers), the average number of nerve fibers (NF-positive fibers) and myelinated nerve fibers (MBP-positive fibers) per 0.04 mm^2^, and the dimensions of myelinated fibers;

* the differences of values are significant in relation to an autograft, p<0.05.

**Figure 6.**
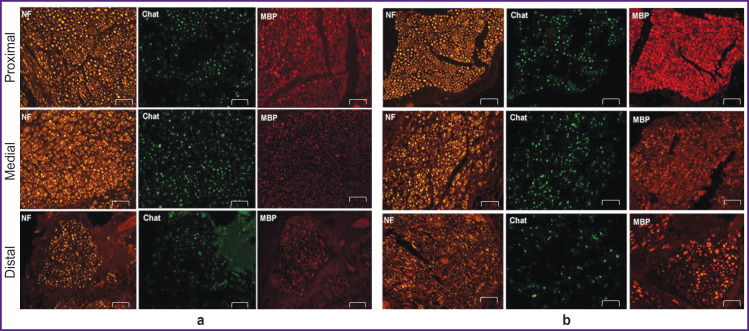
Immunohistochemical assessment of the regeneration efficiency of proximal, medial, and distal segment of the peripheral nerve in rats on day 90 after implantation: (a) autograft; (b) decellularized scaffold; 100×

After staining by the antibody to MBP the nerve fibers were divided into two types. The first type consisted of small fibers, 1–6 μm in size, which indicated the nerve regeneration. The second type was characterized by large fibers, 7–20 μm in size, which were likely to have already been present before the experiment. Figure 6 shows there was a well-defined increase in the number of small fibers in medial nerve segments compared to other nerve segments. Moreover, the number of ChAT-positive fibers in the total number of nerve fibers stained positively for neurofilaments (NF-positive fibers) preserved in balance with 40% limit for ChAT. Finally, motor and sensory fibers were found to restore. All samples appeared to have an increase in the number of small myelinated fibers in the medial segment. Thus, the findings demonstrated high regenerability of the experimental material in the peripheral nerve damage in rats.

## Discussion

A number of studies [11–15] have confirmed both — the efficiency and the safety of using decellularized nerve scaffolds when restoring peripheral nerve injuries that makes them a promising alternative to autografting. The main advantage of decellularized nerves is their high compatibility, which is due to a low content of a cellular component. In addition, they preserve optimal characteristics of mechanical strength and elasticity. The presence of basic structural components of ECM and the preserved three-dimensional structure promote a regenerative process in damaged peripheral nerves [16].

The most common decellularization method is the combination of detergents with enzymatic treatment. Zwitterionic and nonionic detergents: sulfobetaine-10 (SB-10), sulfobetaine-16 (SB-16), and Triton X-100 are frequently used to solve nuclear, cellular membranes and cytoplasmic proteins. The study by Zaminy et al. [15] suggested a protocol for producing decellularized nerve scaffolds using the combination SB-10, SB-16, and Triton X-100, which provided a high level of cell elimination, the preserved structure of collagen nerve fibers and no cytotoxicity. Quantitative DNA analysis showed a decellularized nerve scaffold to contain DNA by 93.5% less than the native samples, and MTT assay revealed no cytotoxicity signs. Our protocol for nerve decellularization involved using the combination of Triton X-100 and the enzymatic treatment. The comparative analysis of the DNA amount left after treatment showed the decellularized scaffold samples to contain DNA by 78.25% less compared to the native samples; moreover, the obtained result corresponded to the recognized criterion of decellularization quality (~50 ng/mg of tissue) [17]. One more substantial difference of the protocols under comparison was the treatment time. The cycle of obtaining decellularized samples in Zaminy et al. [15] study lasted 62 h, whereas in our protocol the treatment time was 22 h. Thus, the decellularized nerve scaffolds obtained using our protocol and the protocol resulted from the study by Zaminy et al. [15] can be effectively applied to restore damaged peripheral nerves.

Decellularization using only Triton X-100 is not as effective for DNA removal as the combination of different detergents. So, Topuz and Aydin [18] undertook the comparative analysis of detergent combinations and concluded that the treatment of nerve samples with Triton X-100 and sodium desoxycholate to enable to achieve an optimal result. The DNA analysis in the decellularized nerve showed the less amount of DNA than in the native nerves, no higher cytotoxicity of the decellularized scaffold being revealed. Our protocol also envisaged the use of Triton X-100 combined with sodium desoxycholate. The comparative analysis of DNA content in the samples of the native nerve and the decellularized one demonstrated such combination of detergents to significantly decrease the content of cellular components.

In the research by Wang et al. [19], the decellularized nerve was obtained by three different decellularization techniques, and two of them included the enzymatic treatment with DNase I. The comparative findings of the decellularization protocols showed the optimal removal of cellular components after enzymatic treatment. However, the method including the enzymatic treatment with the preliminary nerve freezing/unfreezing appeared to be more effective than the two other decellularization methods with chemical extraction. Our decellularization protocol included the stage of DNase I, although the treatment time was three times less than in the study by Wang et al. [19]. The histological analysis showed the presence of nuclei and ordered collagen fibrils in the native nerve samples. The decellularized nerve samples obtained using a detergent-enzymatic method according to our protocol were characterized by the less number of nuclei and the presence of fiber breaking. In decellularization according to the protocol by Wang et al. [19] the cellular component amount after decellularization was significantly lower, and collagen fibers preserved well and were arranged parallel to each other.

The study by Contreras et al. [20] involved the efficacy evaluation of the defect restoration (15 mm) of the sciatic nerve in rats. The decellularized nerve scaffold demonstrated promising results in axon regeneration. The histological examination revealed the optimal integrity of ECM structure, as well as the presence of numerous regenerated myelinated and unmyelinated axons in the medial and distal segments of the decellularized scaffold after implantation. Immunohistochemical staining demonstrated the presence of numerous axons surrounded by Schwann cells along the decellularized nerve. The decellularized scaffold had slower regeneration, and Schwann cells were loosely located compared to the autograft. In our study the use of the decellularized nerve scaffold contributed to the restoration of an extensive peripheral nerve damage in rats. The scaffold had three-dimensional mesh structure of native nerves, which set the direction of axonal growth. The immunohistochemical and histological staining findings demonstrated the decellularized nerve scaffold ability to stimulate the regenerative processes of motor and sensory fibers in damaged peripheral nerves in rats. Additionally, there was found the increase in the number of small myelinated fibers in the medial segment and the moderate number of damaged nerve fibers throughout the scaffold.

## Conclusion

The use of detergent-enzymatic decellularization of the rat sciatic nerve provided the effective removal of cellular components, the integrity preservation of the endoneurium and the main ECM components. The decellularized scaffold of the peripheral nerve resulted in neither rejection nor inflammatory infiltration in hetero-and orthotopical implantation. The decellularized scaffold of the peripheral nerve is a promising alternative to using autografts in replacing extensive defects of peripheral nerves.

## References

[1] Jahromi M., Razavi S., Bakhtiari A (2019). The advances in nerve tissue engineering: from fabrication of nerve conduit to in vivo nerve regeneration assays.. J Tissue Eng Regen Med.

[2] Boriani F., Fazio N., Bolognesi F., Pedrini F.A., Marchetti C., Baldini N (2019). Noncellular modification of acellular nerve allografts for peripheral nerve reconstruction: a systematic critical review of the animal literature.. World Neurosurg.

[3] Brown M., Li J., Moraes C., Tabrizian M., Li-Jessen N.Y.K (2022). Decellularized extracellular matrix: new promising and challenging biomaterials for regenerative medicine.. Biomaterials.

[4] Zhang X., Chen X., Hong H., Hu R., Liu J., Liu C (2021). Decellularized extracellular matrix scaffolds: recent trends and emerging strategies in tissue engineering.. Bioact Mater.

[5] Ikegami Y., Ijima H (2021). Decellularization of nervous tissues and clinical application.. Adv Exp Med Biol.

[6] Wüthrich T., Lese I., Haberthür D., Zubler C., Hlushchuk R., Hewer E., Maistriaux L., Gianello P., Lengelé B., Rieben R., Vögelin E., Olariu R., Duisit J., Taddeo A (2020). Development of vascularized nerve scaffold using perfusion-decellularization and recellularization.. Mater Sci Eng C Mater Biol Appl.

[7] Xu S., Lu F., Cheng L., Li C., Zhou X., Wu Y., Chen H., Zhang K., Wang L., Xia J., Yan G., Qi Z (2017). Preparation and characterization of small-diameter decellularized scaffolds for vascular tissue engineering in an animal model.. Biomed Eng Online.

[8] Gilpin A., Yang Y (2017). Decellularization strategies for regenerative medicine: from processing techniques to applications.. Biomed Res Int.

[9] Philips C., Cornelissen M., Carriel V (2018). Evaluation methods as quality control in the generation of decellularized peripheral nerve allografts.. J Neural Eng.

[10] (2010). GOST ISO 10993-5—2011. Izdeliya meditsinskie. Otsenka biologicheskogo deystviya meditsinskikh izdeliy. Chast’ 5. Issledovaniya na tsitotoksichnost’: metody in vitro [GOST ISO 10993-5—2011. Medical products. Assessment of the biological effects of medical devices. Part 5. Cytotoxicity studies: in vitro methods]..

[11] Nasrollahi Nia F., Asadi A., Zahri S., Abdolmaleki A (2020). Biosynthesis, characterization and evaluation of the supportive properties and biocompatibility of DBM nanoparticles on a tissue-engineered nerve conduit from decellularized sciatic nerve.. Regen Ther.

[12] Kuna V.K., Lundgren A., Anerillas L.O., Kelk P., Brohlin M., Wiberg M., Kingham P.J., Novikova L.N., Andersson G., Novikov L.N (2022). Efficacy of nerve-derived hydrogels to promote axon regeneration is influenced by the method of tissue decellularization.. Int J Mol Sci.

[13] McCrary M.W., Vaughn N.E., Hlavac N., Song Y.H., Wachs R.A., Schmidt C.E (2020). Novel sodium deoxycholate-based chemical decellularization method for peripheral nerve.. Tissue Eng Part C Methods.

[14] Nakada M., Itoh S., Tada K., Matsuta M., Murai A., Tsuchiya H (2020). Effects of hybridization of decellularized allogenic nerves with adipose-derive stem cell sheets to facilitate nerve regeneration.. Brain Res.

[15] Zaminy A., Sayad-Fathi S., Kasmaie F.M., Jahromi Z., Zendedel A (2021). Decellularized peripheral nerve grafts by a modified protocol for repair of rat sciatic nerve injury.. Neural Regen Res.

[16] Philips C., Campos F., Roosens A., Sánchez-Quevedo M.D.C., Declercq H., Carriel V (2018). Qualitative and quantitative evaluation of a novel detergent-based method for decellularization of peripheral nerves.. Ann Biomed Eng.

[17] Crapo P.M., Gilbert T.W., Badylak S.F (2011). An overview of tissue and whole organ decellularization processes.. Biomaterials.

[18] Topuz B., Aydin H.M (2022). Preparation of decellularized optic nerve grafts.. Artif Organs.

[19] Wang Q., Zhang C., Zhang L., Guo W., Feng G., Zhou S., Zhang Y., Tian T., Li Z., Huang F (2014). The preparation and comparison of decellularized nerve scaffold of tissue engineering.. J Biomed Mater Res A.

[20] Contreras E., Traserra S., Bolívar S., Forés J., Jose-Cunilleras E., García F., Delgado-Martínez I., Holmgren S., Strehl R., Udina E., Navarro X (2022). Repair of long nerve defects with a new decellularized nerve graft in rats and in sheep.. Cells.

